# Anodal transcranial direct current stimulation reduces motor slowing in athletes and non-athletes

**DOI:** 10.1186/s12868-020-00573-5

**Published:** 2020-06-01

**Authors:** Oliver Seidel-Marzi, Patrick Ragert

**Affiliations:** 1 Institute for General Kinesiology and Exercise Science, Faculty of Sport Science, University of Leipzig, Jahnallee 59, 04109 Leipzig, Germany; 2 Department of Neurology, Max Planck Institute for Human Cognitive and Brain Sciences, Stephanstraße 1a, 04103 Leipzig, Germany

**Keywords:** tDCS, Motor slowing, Tapping task, Primary motor cortex, Athletes

## Abstract

**Background:**

Motor fatigability describes a phenomenon that occurs when exhaustive exercise or physically demanding tasks are executed over an extended period of time. Concerning fast repetitive movements, it is noticeable by a reduction in movement speed (motor slowing, MoSlo) and occurs due to both central and peripheral factors. The aim of the present study was to examine the presence of MoSlo during hand- (HTT) and foot-tapping tasks (FTT) comparing trained football (FB) and handball players (HB) and non-athletes (NA). Furthermore, we were interested in how far anodal transcranial direct current stimulation (tDCS) might be capable of modulating MoSlo as compared to sham.

**Methods:**

A total number of 46 participants were enrolled in a sham-controlled, double-blinded, cross-over study. HTT and FTT were performed before, during, after as well as 30 min after 20 min of tDCS over the leg area of the primary motor cortex (M1).

**Results:**

We could demonstrate that MoSlo during HTT and FTT is a general phenomenon that is observed independent of the type of sports and/or training status. Furthermore, we were able to show a tDCS-induced reduction in MoSlo specifically during FTT in both trained athletes and NA. No such effects could be observed for HTT, indicating local specificity of tDCS-induced effects on a behavioral level.

**Conclusion:**

We could demonstrate that tDCS is capable of reducing motor fatigability during fast repetitive movements. These findings are of pivotal interest for many sports where fatigability resistance is a limiting factor in maintaining repetitive movement patterns.

## Background

Motor fatigability describes a phenomenon that occurs when exhaustive exercise or physically demanding tasks are executed over an extended period of time. There is compelling evidence that motor fatigability, that is defined as the exercise-dependent decrease in the ability of muscle fibers to generate force or power [1] and that is used to refer to objective changes in performance [2], occurs due to both central and peripheral factors [3]. While most fatigability occurs within the muscle (peripheral fatigue), some fatigability can also be attributed to voluntary activation of the muscle declines (central fatigue), or reflects suboptimal output from the motor cortex (supraspinal fatigue) [4–7]. According to a recent review [8], central and supraspinal fatigue might be related to a decrease in the central command, particularly during exercises of long duration and low intensity [5, 9]. More specifically, it is mediated by the activity of cerebral neurotransmitters and muscular afferent fibers [8, 10]. Peripheral fatigue, however, is described as an impairment of mechanisms from excitation to muscle contraction [8]. Here, phosphate accumulation, perturbation of calcium ion movements, and/or decreases of adenosine triphosphate stores are potential triggers [8, 11]. Although the interaction between central and peripheral mechanisms is described as leading to a series of events that critically affect the muscle’s capacity of generating force [8], current knowledge of the neurophysiological mechanisms underlying motor fatigability still remains elusive.

Indeed, motor fatigability is a complex, multifactorial phenomenon whose mechanisms are influenced by the characteristics of the task being performed [12]. Concerning muscle exercise, fatigability is defined as any exercise induced decrease in maximal voluntary force or power [13] and the inability to maintain the required level of strength [14]. Previous studies reported the presence of motor fatigability during isometric contraction of several muscles such as foot flexors [9], ankle dorsiflexors [15, 16] or elbow flexors [4, 5] using different intensities of maximal voluntary contraction (MVC). Moreover, motor fatigability in quadriceps muscles has been investigated in several previous studies since this muscle group is relevant especially for locomotor movements [17, 18]. Taken together, these studies suggest that motor fatigability appears to contribute significantly to the decrease in force generation during low-intensity exercise. Even more interestingly, brain stimulation techniques such as transcranial direct current stimulation (tDCS) seem to be capable of modulating motor fatigability by manipulating brain excitability of related motor cortical areas. tDCS produces a noninvasive electrical stimulus that promotes changes in the resting potential of the neuronal membrane [19]. While anodal tDCS induces an increase of area-specific excitability, cathodal tDCS induces opposing effects [19], which can be observed by changes in the motor evoked potential (MEP) evoked by transcranial magnetic stimulation (TMS). For example, Cogiamanian et al. [20] investigated potential tDCS-induced effects on fatigability during a submaximal isometric contraction of left elbow flexors while stimulating the right primary motor cortex (M1). Their results indicate that anodal tDCS led to a reduction in muscle fatigability. These findings were confirmed by a recent systematic review and meta-analysis by Lattari et al. [21] concerning acute effects of single dose of tDCS on muscle strength, suggesting that the use of tDCS may promote increases in maximal voluntary contraction and muscular endurance through isometric contractions in novice and advanced strength training. Taking this into account, Banissy and Muggleton [22] assume that it is possible to modulate fatigability to a large degree with tDCS stimulation. While there is some controversy about tDCS-induced effects on motor performance [23], recent reviews suggest that tDCS may have a moderate positive impact on performance levels [24, 25].

Apart from isometric muscle exercise, knowledge about motor fatigability during fast repetitive movements is rather sparse. In this context, motor fatigability is often referred to as motor slowing (MoSlo). In finger tapping tasks, MoSlo has been observed as a characteristic reduction of movement speed. However, MoSlo also occurs during skilled motor tasks such as motor sequence tapping involving multiple fingers [26], and during tapping at the maximal voluntary rate (MVR) for a short period of time [27–29]. These studies suggest that movement speed drops in a few seconds when tapping is performed at MVR. Using repetitive TMS (rTMS), findings of Jäncke et al. [30] indicate that M1 is essential for generating fastest finger movements. In detail, they demonstrated that rTMS of the left M1 slowed finger tapping speed of the right hand during tapping at MVR. Revealing this target region, tDCS delivered over M1, which has been demonstrated to be capable of modulating the excitability of this region in several studies [19], seems to be a promising method to investigate possible effects on MoSlo. Transferred into a context of training and competitive sports, these findings might be of pivotal interest since fatigability resistance is a limiting factor in many sports [31] with regards to repetitive movement patterns.

Hence, the primary aim of the present study was to examine the presence of MoSlo as a decline in tapping frequency during hand- (HTT) and foot-tapping (FTT) tasks. The focus was on the question whether MoSlo differs between trained athletes and non-athletes and to what extend the decline can be modulated by means of tDCS. First, we expected the tapping frequency to decrease in both upper and lower extremities [32], regardless of training status and sports. On an exploratory level, we aimed at revealing if athletes would show specific MoSlo patterns as compared to non-athletes [33], and, furthermore, if there are any differential effects between different kinds of sport. Therefore, we recruited athletes from predominantly hand- and foot-dominant sports (handball and football) to pursue this question. Furthermore, we hypothesized that anodal tDCS over M1 (leg area) might be capable of reducing MoSlo during FTT (not HTT, since this can be considered as a kind of control condition) as compared to sham condition. This hypothesis was motivated by previous studies demonstrating that anodal tDCS over M1 leg area enhances leg motor cortex excitability bilaterally [34–36]. On a behavioral level, Kaminski et al. [37] provided novel evidence for the ability of anodal tDCS over M1 leg area to improve dynamic balance performance in the lower limb and Tanaka et al. [38] were even able to demonstrate that anodal tDCS transiently enhanced the maximal leg pinch force without affecting hand pinch force, showing the spatial specificity of the effect of tDCS.

## Materials and methods

Descriptions made below are based on a previously published dataset we acquired [39]. While participants and the experimental design are equivalent to this previous work, the present study focused on a novel research question investigating the effects of tDCS-induced modulations of motor slowing. For further details on experimental setup, please refer to Seidel and Ragert [39].

### Ethical approval

The study was approved by the local ethics-committee of the Medical Faculty at the University of Leipzig. All participants gave written informed consent to participate in the experiments according to the Declaration of Helsinki.

### Participants

In the present study, a total number of 46 healthy, young adults were recruited from the database of the Max-Planck-Institute for Human Cognitive and Brain Sciences as well as through public advertisement. The investigated sample of this study consisted of 13 football players (FB, three females, age = 24.00 ± 3.89 years (mean ± SD)), 12 handball players (HB, five females, age = 22.50 ± 4.32 years) and 21 non-athletes (NA, 11 females, age = 26.95 ± 3.43 years). Inclusion criteria for FB and HB involved an individual training history of at least 2 years and regular practice and participations in competitions/matches in their respective sports discipline. NA were not allowed to do more than 2 h of combined sports activities (any specific physical activity outside of their daily routine) per week. On average, FB trained for 16.31 ± 5.02 years and currently 5.65 ± 2.15 h/week, whereas HB trained for 13.17 ± 4.49 years and currently 8.54 ± 3.84 h/week. On the other hand, NA performed less than 2 h of combined sports activities per week (1.41 ± 1.32 h/week). All participants were right-handed (mean laterality quotient (LQ) ± SD, FB: 84.02 ± 16.45; HB: 95.83 ± 8.14; NA: 90.15 ± 14.15) according to the Oldfield handedness inventory [40] and none of them had any history of playing musical instruments. Additionally, all participants underwent a detailed neurological examination and were instructed to avoid alcohol and caffeine intake 24 h prior to testing [41].

### Experimental design

**Fig. 1:**
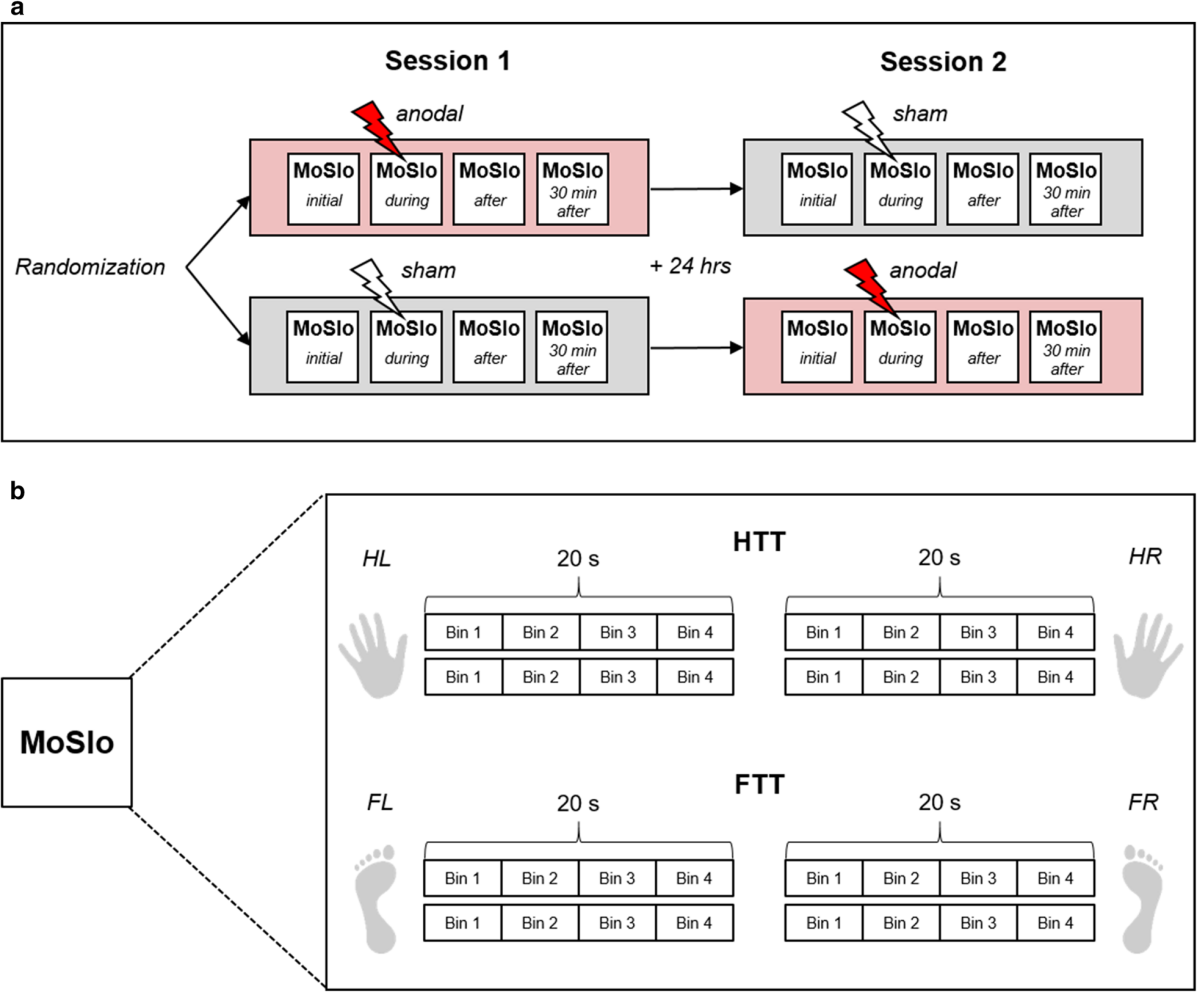
Study design and experimental setup. **a** Procedures for session 1 and 2. Study procedure for both sessions was identical, starting with an *initial* run of the test block *MoSlo* (motor slowing) containing hand tapping tasks (HTT) for left (HL) and right (HR) hand and foot tapping tasks (FTT) for left (FL) and right (FR) foot. Afterwards, transcranial direct current stimulation (tDCS) was applied over the leg area of the primary motor cortex (M1 leg area) for a period of 20 min, symbolized by the lightning. Participants were randomly assigned to one stimulation type, receiving either the anodal tDCS condition (red lightning) or the control condition (white lightning), where sham tDCS was applied (the other condition was applied in session 2, accordingly). Another run of HTT and FTT was performed after 10 min of stimulation (*during* tDCS, online) as well as directly *after* and *30 min after* stimulation has ended (offline). **b** Behavioral tasks. Participants were asked to perform four runs of the test block *MoSlo* (*initial*, *during*, *after* and *30* *min after* tDCS), each consisting of two runs of a 20-second HTT and FTT for each hand and foot separately. Tapping frequency was analyzed in 5-second bins

We used a sham-controlled, double-blinded, cross-over design to apply two conditions of tDCS (anodal, sham) to the bilateral M1 leg area while participants performed a 20-second tapping task either with their upper (HTT) or lower (FTT) extremities. The study was compromised of two sessions that were separated by at least 24 h to avoid confounding effects of central and peripheral fatigue on subsequent performances. Study procedure for both sessions was identical (Fig. 1a), starting with an *initial* run of a test block of HTT and FTT. Afterwards, tDCS was applied for a period of 20 min. Participants received either the anodal tDCS condition or the control condition, where sham tDCS was applied. For each participant, the type of stimulation was randomly assigned to either session 1 or 2. Another run of the aforementioned test block was performed after 10 min of stimulation (*during* tDCS, online) as well as directly *after* and *30 min after* stimulation has ended (offline).

### Hand- (HTT) and foot-tapping (FTT) tasks

For our experimental task, all participants were instructed to maintain an upright position on a stool with both of their hands resting comfortably on and their feet resting under a table with a defined distance of 10 cm to four custom-made force plates. Further details of the experimental setup can be found elsewhere [39].

Participants performed four runs of a test block (*initial*, *during*, *after* and *30 min after* tDCS), each consisting of two runs of HTT for left (HL) and right (HR) hand and FTT for left (FL) and right (FR) foot, respectively (Fig. 1b). Before each run, the upcoming task appeared on the computer monitor followed by a countdown of 3 s. Afterwards, participants started the run on their own with their first touch of the respective force plate. Subsequently, they had to touch the force plate as often as possible over a period of 20 s. Concerning HTT, participants were instructed to tap in the center of the force plate with a flat hand. For FTT, they were asked to keep the heel up in the air and to tap with their forefoot. As an outcome measure, tapping frequency (Hz) was recorded.

### Transcranial direct current stimulation (tDCS)

**Fig. 2:**
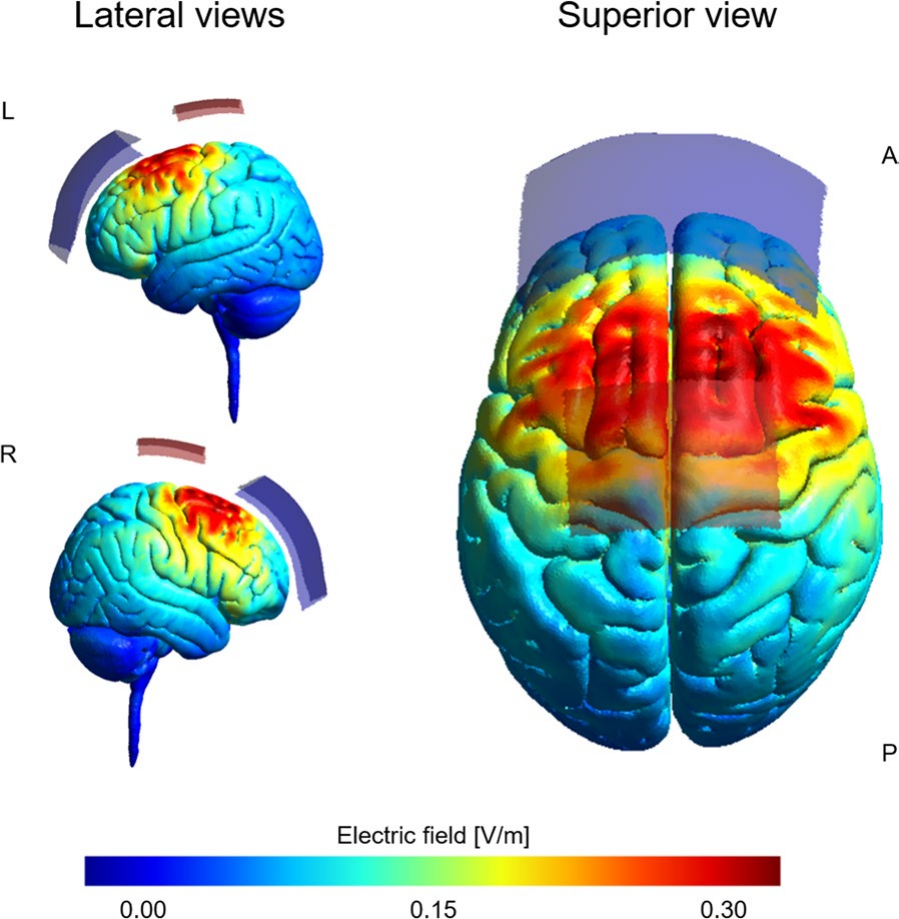
tDCS montage and electric field distribution modelling. Figure illustrates electric field distribution of the applied tDCS setup on the MNI head model using an intensity of 2 mA. The electrodes were placed according to the 10–20 system of a standard 64-channel electroencephalography (EEG) cap with the anode (7 cm × 5 cm, size = 35 cm^2^) positioned over Cz and the cathode (reference electrode, 10 cm x 10 cm, size = 100 cm^2^) over the middle of the forehead (Fz). Electrodes are indicated as a red (anode) or blue (cathode) shade. The left part of the figure provides lateral views of the brain from left (L) and right (R), while the right part displays the superior view (*A* anterior, *P* posterior)

tDCS was delivered by a battery driven-stimulator (neuroConn GmbH, Ilmenau, Germany) using a pair of surface electrodes in saline-soaked (0.9% NaCl) synthetic sponges and flexible elastic straps to fixate the electrodes on the head. For each session, either anodal tDCS or sham tDCS was applied to the leg area of M1, stimulating both left and right M1 simultaneously (for tDCS current field modelling see Fig. 2). While the anode (7 cm × 5 cm, size = 35 cm^2^) was placed over Cz (M1 leg area target region), the cathode (reference electrode, 10 cm × 10 cm, size = 100 cm^2^) was placed over the middle of the forehead (Fz). The current was ramped up for 30 s at the beginning of tDCS eliciting a transient tingling sensation on the scalp that faded over seconds [42, 43] and also ramped down for 30 s. During anodal and sham conditions the current was applied with an intensity of 2 mA for 20 min, whereas during the sham condition stimulation lasted 30 s and subsequently ramped down to no stimulation. Researchers, as well as participants, were blinded during the experiments. Immediately after the electrodes were removed, participants were asked to report potential unpleasant side effects due to tDCS stimulation such as tingling sensations, burning, itching/scratching sensations and headache/pain. Further details of the tDCS procedure can be found elsewhere [39].

To verify the selected tDCS setup for the stimulation of M1 leg area, we performed a modelling of the electric field distribution of the mentioned electrode setup (Fig. 2) using SimNIBS 3.0.6 [44] and the included MNI head model. Current field modelling was conducted using the standard SimNIBS pipeline and previously established conductivity values for each tissue type according to Opitz et al. [45]: white matter (WM, 0.126 Siemans/meter (S/m)), gray matter (GM, 0.275 S/m), cerebrospinal fluid (CSF, 1.654 S/m), bone (0.010 S/m) and skin (0.465 S/m). The anode (size = 35 cm^2^) was placed over Cz and the cathode (size = 100 cm^2^) was positioned over Fz, using positions based on the 10-20 system of a standard 64-channel electroencephalography (EEG) cap. Electrodes were modeled to represent those produced by neuroConn [46]. The electric field distribution modelling revealed that with the applied montage in fact both hemispheres were stimulated, presumably the leg area of M1 (among other regions such as SMA) and parts of M1 hand area (for details see Fig. 2).

### Analysis

Two runs of HTT and FTT were recorded for each test block for HL, HR, FL and FR, respectively. Tapping time of 20 s was divided into 4 bins of 5 s and averaged for both runs of one test block. This resulted in 4 frequency values (bin 1 = 0–5 s, bin 2 = 5–10 s, bin 3 = 10–15 s and bin 4 = 15–20 s) before (*initial*), *during*, *after* and *30 min after* tDCS stimulation. Baseline differences were tested using a univariate ANOVA and revealed significant differences between groups. Hence, values were normalized to the first bin of the first test block (*initial*_0–5_ = 100%). In a final step, the presence of MoSlo was defined as a reduction of tapping frequency (deltas) from the first to the last bin of the normalized data of each test block [32].

All statistical analyses were performed with the software SPSS 25 (IBM, Armonk, NY, USA) using parametric tests since Shapiro–Wilk test revealed that HTT and FTT data were normally distributed. As already described above, baseline differences were examined using a univariate ANOVA with factor group (FB vs. HB vs. NA) using Gabriel and Games-Howell post hoc tests, respectively, to analyze the differences if necessary. First, the presence of MoSlo was examined using the *initial* test block in session 1 of each participant. Therefore, a 3 × 3 repeated measures ANOVA was conducted to analyze the mean normalized frequency values of each group and each extremity for three bins of HTT and FTT (within-subject factor), including group (FB vs. HB vs. NA) as between-subject factor. The first bin was not included since data were normalized and level 0–5 would not have any variance across participants since all of them would have a value of 100%. Second, in order to reveal tDCS-induced effects on MoSlo, a 2 × 3 × 4 repeated measures ANOVA was conducted to analyze the mean delta values of each group and each extremity for four test blocks of HTT and FTT (first within-subject factor), including stimulation condition (anodal vs. sham) as second within-subject factor and group (FB vs. HB vs. NA) as between-subject factor.

When the respective interactions were significant, also Gabriel and Games-Howell post hoc tests, respectively, were applied to analyze the differences. The critical level of significance in all tests was set to p < 0.05 and Bonferroni-adjusted for multiple comparisons. If necessary, data were corrected for sphericity using Greenhouse–Geisser correction. Partial eta-squared ($\eta_{p}^{2}$) for ANOVAs are provided as measures of effect size and used to aid in the interpretation of inferential statistics. As a rule of thumb, introduced by Miles and Shevlin [47], $\eta_{p}^{2}$ ≥ 0.01 is considered to be a small, $\eta_{p}^{2}$ ≥ 0.06 a medium, and $\eta_{p}^{2}$ ≥ 0.14 a large effect. Additionally, as recommended for tDCS studies by Biel and Friedrich [48], Bayes factors (BF), a useful tool for evaluating evidence both for the research hypothesis and for the null hypothesis [49, 50], are reported for repeated measures ANOVAs using JASP (Jeffreys’s Amazing Statistics Program [51]). BFs above 1 indicate evidence for H1 over H0, whereas BFs below 1 suggest the exact opposite. If BFs are above 3 or below 0.33, the strength of evidence for one hypothesis compared to its competing hypothesis is regarded as noteworthy [52, 53]. Thus, BFs between 0.33 and 3 are considered as inconclusive, or only anecdotal evidence for any hypothesis.

## Results

### Baseline comparisons

Baseline comparisons of bin 1 revealed significant differences between groups indicating higher values in FB and HB compared to NA. uANOVA showed a significant main effect of group in HL (F_(2,43)_ = 12.081, p = 0.000, $\eta_{p}^{2}$ = 0.360), HR (F_(2,43)_ = 11.268, p = 0.000, $\eta_{p}^{2}$ = 0.344), FL (F_(2,43)_ = 17.144, p = 0.000, $\eta_{p}^{2}$ = 0.444) and FR (F_(2,43)_ = 11.635, p = 0.000, $\eta_{p}^{2}$ = 0.351). Post hoc analyses exposed significant differences between FB and NA in HL (p_adjusted_ = 0.000), HR (p_adjusted_ = 0.000), FL (p_adjusted_ = 0.000) and FR (p_adjusted_ = 0.000) as well as between HB and NA in HL (p_adjusted_ = 0.001), HR (p_adjusted_ = 0.033), FL (p_adjusted_ = 0.000) and FR (p_adjusted_ = 0.003). However, there were no significant differences between FB and HB (HL: p_adjusted_ = 0.987; HR: p_adjusted_ = 0.247; FL: p_adjusted_ = 0.631; FR: p_adjusted_ = 0.851).

### MoSlo during HTT and FTT

**Fig. 3:**
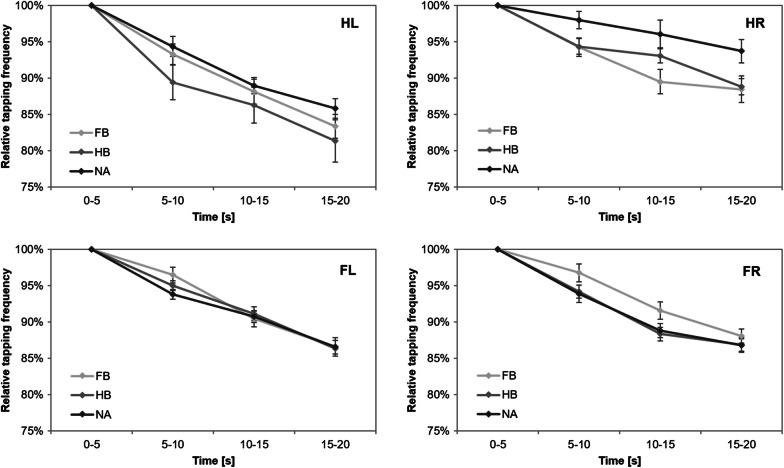
Baseline comparison of motor slowing during HTT and FTT. Diagrams include normalized (% of bin 1) tapping frequency values (mean ± SE) of the *initial* test block of left hand (HL), right hand (HR), left foot (FL) and right foot (FR), respectively. Light gray lines represent football players (FB), medium gray lines represent handball players (HB) and dark gray lines represent non-athletes (NA). Results indicate a significant reduction in tapping frequency in all extremities

Concerning the first test block of each participant, rmANOVA revealed non-significant time x group interactions for HL (F_(4,86)_ = 0.951, p = 0.439, $\eta_{p}^{2}$ = 0.042, BF = 1.624), HR (F_(4,86)_ = 1.378, p = 0.248, $\eta_{p}^{2}$ = 0.060, BF = 0.239), FL (F_(3.218,69.181)_ = 1.875, p = 0.138, $\eta_{p}^{2}$ = 0.080, BF = 0.528) and FR (F_(3.232,69.486)_ = 0.548, p = 0.664, $\eta_{p}^{2}$ = 0.025, BF = 0.115). However, factor time was significant for HL (F_(2,86)_ = 93.420, p = 0.000, $\eta_{p}^{2}$ = 0.685, BF = 1.114e + 20), HR (F_(2,86)_ = 27.270, p = 0.000, $\eta_{p}^{2}$ = 0.388, BF = 1.129e + 7), FL (F_(1.609,69.181)_ = 137.343, p = 0.000, $\eta_{p}^{2}$ = 0.762, BF = 9.852e + 24) and FR (F_(1.616,69.486)_ = 83.089, p = 0.000, $\eta_{p}^{2}$ = 0.659, BF = 9.264e + 18), indicating significant decreases in tapping frequency from bin 1 to 4 (Fig. 3). Post hoc comparisons revealed significant differences between all bins (all p_adjusted_ ≤ 0.006). Moreover, we found a significant influence of factor group only for HR (F_(2,43)_ = 4.058, p = 0.024, $\eta_{p}^{2}$ = 0.159, BF = 3.229), indicating differences in MoSlo between FB and NA (p_adjusted_ = 0.030).

### tDCS-induced effects on MoSlo

In a first step, the success of blinding was evaluated based on the participants’ reports regarding potential side effects due to tDCS procedure. Across all 46 participants, a total number of 32 participants (69.6%) reported mild sensations such as tingling, burning, itching/scratching or headache at the beginning and in the mid-phase of stimulation period for both anodal and sham session. Moreover, 5 participants (10.9%) reported such mild sensations only for anodal tDCS but not for sham condition, whereas for only 2 participants (4.3%) it was vice versa, reporting mild sensations only for sham but not for anodal tDCS. The remaining 7 participants (15.2%) reported no sensations, neither for anodal nor for sham tDCS. Furthermore, no sensations were reported that persisted until the end of stimulation period, as well as no strong sensations or discomfort. Results indicate that the applied blinding procedures were successful.

**Fig. 4:**
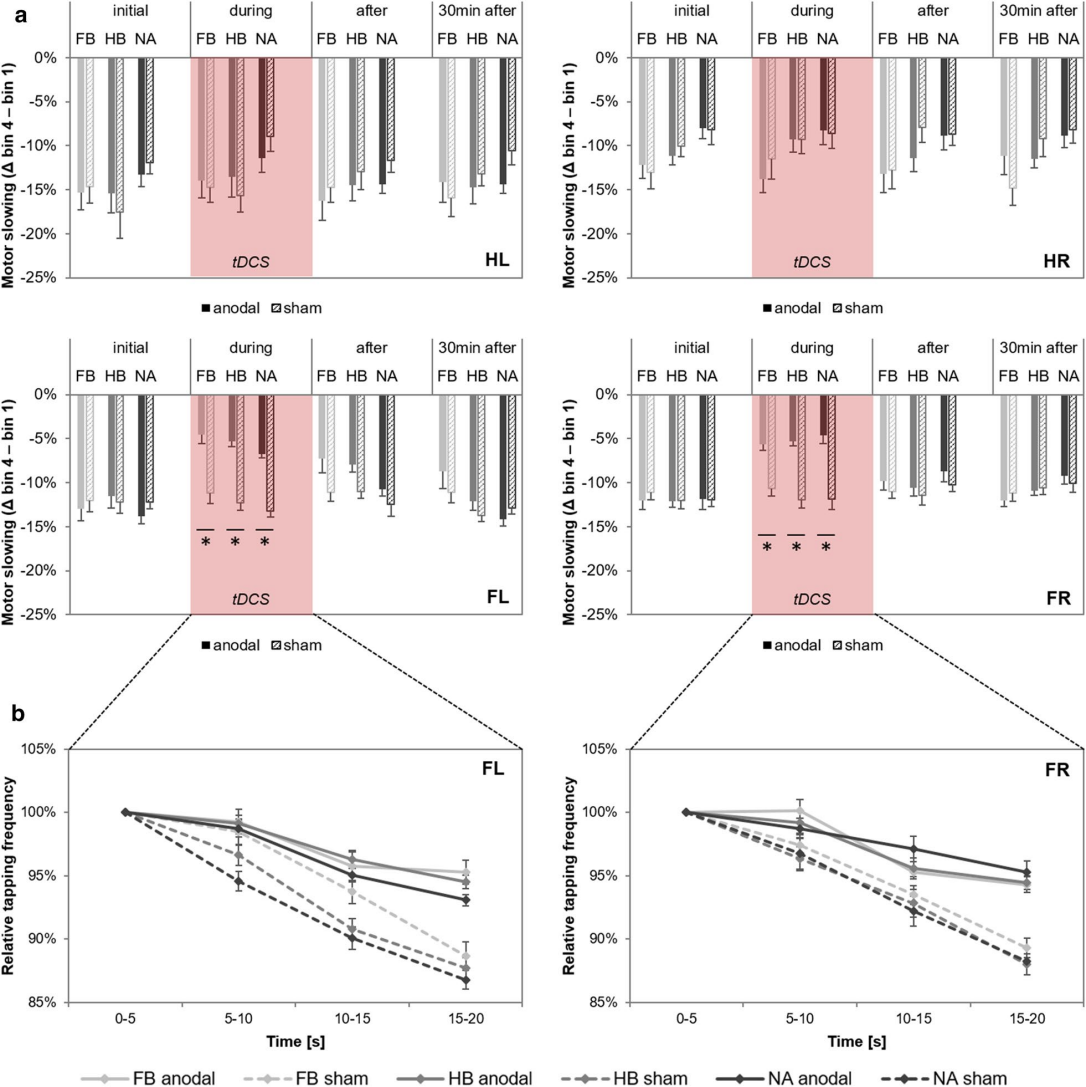
tDCS-induced effects on motor slowing. **a** Decline in tapping frequency for each test block. Values (mean ± SE) are deltas between bin 1 and bin 4 frequency (motor slowing) of left hand (HL), right hand (HR), left foot (FL) and right foot (FR), respectively for before (*initial*), *during*, *after* as well as *30* *min after* a 20-min tDCS application which is indicated by the red box. Light gray bars represent football players (FB), medium gray bars represent handball players (HB) and dark gray bars represent non-athletes (NA). Solid bars define values for anodal tDCS, corresponding dashed bars indicate values for sham tDCS. Results indicate a significant reduction in motor slowing for left and right FTT *during* anodal tDCS as compared to sham tDCS. No effect was found for HTT. **b** tDCS-induced effect on FTT during stimulation. Diagrams include normalized (% of bin 1) tapping frequency values (mean ± SE) of the test block *during* anodal/sham stimulation of left foot (FL) and right foot (FR), respectively. Light gray lines represent football players (FB), medium gray lines represent handball players (HB) and dark gray lines represent non-athletes (NA). Results indicate a significant reduction in motor slowing for left and right FTT *during* anodal tDCS as compared to sham tDCS

Using the decline in tapping frequency of each test block (delta_bin4-bin1_) as an indicator of MoSlo, we investigated possible tDCS-induced effects before (*initial*), *during*, *after* and *30* *min after* stimulation. rmANOVA revealed non-significant time x group x condition interactions for HL (F_(5.176,111.292)_ = 0.682, p = 0.643, $\eta_{p}^{2}$ = 0.031, BF = 0.061), HR (F_(6,129)_ = 1.552, p = 0.167, $\eta_{p}^{2}$ = 0.067, BF = 0.150), FL (F_(6,129)_ = 0.895, p = 0.501, $\eta_{p}^{2}$ = 0.040, BF = 0.086) and FR (F_(6,129)_ = 0.320, p = 0.925, $\eta_{p}^{2}$ = 0.015, BF = 0.036). However, we found significant time x condition interactions for FL (F_(3,129)_ = 30.517, p = 0.000, $\eta_{p}^{2}$ = 0.415, BF = 7.077e + 11) and FR (F_(3,129)_ = 36.106, p = 0.000, $\eta_{p}^{2}$ = 0.456, BF = 4.669e + 13), indicating significantly reduced MoSlo *during* anodal tDCS as compared to sham condition (Fig. 4a, b). For FL, post hoc comparisons revealed significant differences between test blocks (*initial* vs. *during*: p_adjusted_ = 0.000, *initial* vs. *after*: p_adjusted_ = 0.000, *during* vs. *30* *min after*: p_adjusted_ = 0.000, *after* vs. *30* *min after*: p_adjusted_ = 0.001) and conditions (anodal vs. sham: p_adjusted_ = 0.000). Similar post hoc results were found for FR, showing significant differences between test blocks (*initial* vs *during*: p_adjusted_ = 0.000, *initial* vs. *after*: p_adjusted_ = 0.001, *initial* vs. *30* *min after*: p_adjusted_ = 0.013, *during* vs. *after*: p_adjusted_ = 0.000, *during* vs. *30* *min after*: p_adjusted_ = 0.000) and conditions (anodal vs. sham: p_adjusted_ = 0.000).

## Discussion

The present study aimed at investigating the presence of MoSlo as a decline in tapping frequency during HTT and FTT, comparing athletes and NA. Moreover, the focus was on the question to what extend MoSlo can be modulated by means of tDCS. In line with previous studies, we could demonstrate that tapping frequency declines in both upper [27] and lower extremities [22]. More interestingly, our findings indicate that anodal tDCS applied over M1 leg area is capable of reducing MoSlo specifically during FTT. Future studies can use these findings to reveal neurophysiological mechanisms underlying MoSlo during fast repetitive movements in order to transfer this knowledge into a sport-specific context.

### MoSlo during HTT and FTT

We hypothesized that tapping frequency decreases in both upper and lower extremities during a 20-second tapping task, independent from training status and sports. In line with previous studies investigating finger tapping at MVR, we can extend these findings by showing that tapping frequency also slowed down during a HTT. Similar to our findings, Rodrigues et al. [27] observed a performance deterioration after the early phase of a 20-second index finger tapping. Using additional electromyography (EMG) recordings, results indicated no loss of force-generating ability related to electrical stimulation of the muscle [29, 54, 55], leading to the assumption that a breakdown of motor control rather than failure of muscle force generation occurs during tapping. Missenard et al. [56] also emphasize the significant role of the central nervous system (CNS) in order to cope with high levels of fatigability, using a strategy to preserve task success in the presence of acute changes in the neuromuscular system. Therefore, the mechanisms underlying the early decline in tapping frequency seem to be central in origin [29], including a reduction in central motor drive.

Regarding the lower extremities, our findings go in line with previous investigations by Bächinger et al. [32], revealing MoSlo during a 30-second alternating FTT. Performing additional analysis concerning the influence of recovery, authors suggest that the mechanism which causes MoSlo appears to fully recover during the subsequent break. The same paradigm was performed while functional magnetic resonance imaging (fMRI) and EMG were assessed during finger tapping. Surprisingly, results revealed that a reduction in tapping frequency was associated on the one hand with an increased coactivation between the agonistic and antagonistic muscle and on the other hand with an increased activation of the motor network (primary sensorimotor cortex (SM1), dorsal premotor cortex (PMd), supplementary motor area (SMA), [57, 58]) which gradually normalized during the subsequent recovery period. According to the authors, the observed increase in excitability in the motor system might be dysfunctional and indicate a breakdown of surround inhibition [59], causing an increase of the excitation-inhibition ratio at the level of M1 towards more net excitation, and thus, leading to a performance deterioration. Therefore, authors suggest that this form of motor fatigability is largely mediated by central mechanisms.

### tDCS-induced reduction of MoSlo during FTT

We expected anodal tDCS over M1 leg area to be capable of reducing MoSlo specifically during FTT. Our results indicate an effecter-specificity of tDCS meaning that tDCS induces changes in leg motor function without affecting hand motor function. According to Tanaka et al. [38], this spatial specificity is presumably possible because the hand motor cortex is about 3–4 cm apart from the leg motor cortex. While tDCS electric field modelling revealed that M1 leg area was not exclusively stimulated in our study (Fig. 2), the behavioral specificity of tDCS effects as shown by Tanaka et al. [38] and in the present study seems to be surprising. While we cannot make direct inferences about the lack of effects for HTT, it seems reasonable to assume that, even though M1 hand area was at least partially stimulated via tDCS over M1 leg area, this modulation did not translate into overt behavioral changes in HTT performance/MoSlo. Hence, future studies should investigate the underlying neural mechanisms of tDCS-induced changes in MoSlo more thoroughly.

There are various reasons for the positive tDCS effects on lower limb function, starting with the ability of anodal tDCS to increase motor cortical excitability [19, 60, 61]. Furthermore, it has been speculated that this could also lead to an increase in supraspinal drive by inducing a prolonged facilitation of corticospinal neurons [20]. In the upper extremity, results of Cogiamanian et al. [20] support this potential mechanism by demonstrating that anodal tDCS applied over the right M1 prolonged endurance time for contralateral elbow flexors in a submaximal isometric task, showing that brain stimulation can modulate motor fatigability. However, other studies also contrast this finding as they found that improvement in motor performance appears not to rely on changes in corticospinal response [62, 63]. Apart from an increased motor cortical excitability, authors assumed further explanations such as widespread tDCS-induced activation changes [64, 65], a decreased fatigue-related muscle pain [66] or an improved synergist muscle coupling [67]. Further explanations would be purely speculative due to the current state of knowledge. However, the exact mechanism is clearly important when attempting to induce benefits of tDCS while minimizing any potential drawbacks [22]. Nevertheless, previous investigations and the present study have demonstrated the possibility to modulate motor fatigability/slowing to a large degree by means of tDCS.

What still remains unsolved is the question in how far tDCS has a beneficial effect in highly trained athletes. While there is convincing evidence that not only training shapes the brain [68–72] but also expertise in a specific sports discipline leads to selective neuroplastic changes on a functional and structural level [73–75], it still remains elusive if athletes are susceptible for tDCS-induced effects [22, 24, 25, 31, 76–78]. Therefore, the aim of the present study was to investigate whether tDCS is capable of evoking changes in motor fatigability in athletes. In fact, it might be reasonable to assume that athletes per se show a kind of ceiling effect in their performance levels which might potentially lead to no detectable tDCS effects on MoSlo. The present findings, however, highlight that motor fatigability can be modulated by tDCS even in trained athletes. Furthermore, we provide preliminary evidence that tDCS-induced effects do not depend on a specific sports discipline since both FB and HB show comparable effects for FTT only.

### Study limitations

In the present study, we used anodal tDCS to modulate the effect of MoSlo in athletes and NA during fast repetitive movements. We were able to demonstrate the presence of MoSlo during HTT and FTT and that anodal tDCS applied over M1 leg area can have specific behavioral effects on FTT. After electric field distribution modelling, we found that tDCS was not as locally specific as expected. Hence, we put into perspective that although M1 hand area was most likely modulated via tDCS, this modulation obviously did not translate into overt behavioral changes in HTT performance. Instead only FTT was altered, a phenomenon that needs to be investigated further in future studies by direct excitability measures using TMS and/or EEG. Moreover, this study was not designed to reveal the neurophysiological mechanisms underlying MoSlo. Therefore, further studies that combine neurophysiological assessments in central (i.e. fMRI, EEG) and peripheral (i.e. EMG) regions with behavioral outcome measures are needed. Additionally, the role of other key regions such as the cerebellum or SMA, which are known to be important for movement sequencing [79], need to be further investigated. Furthermore, the variability and individual responses to tDCS treatment need to be considered as another limiting factor, which has been reported recently [80, 81]. Although our results indicate that tDCS-induced effects on MoSlo are transient, we did not investigate the role of multiple tDCS-sessions on MoSlo. It is known that tDCS also affects consolidation [82] and, it is worth considering that multiple tDCS applications might induce effects that could be more persistent. Clearly, as a further limitation, our study was not designed to disentangle central from peripheral factors on MoSlo. Here, we only showed that modulating brain regions such as M1 by means of tDCS is capable of evoking alterations in MoSlo. However, we cannot exclude the fact that tDCS over M1 leg area also modulated adjacent motor areas and networks which in turn led to a modulation in MoSlo. Additionally, the question remains if such a modulation in MoSlo is primarily driven by supraspinal or central fatigue and should therefore be investigated in future studies.

## Conclusion

We could demonstrate that MoSlo during fast repetitive movements is a general phenomenon that is observed independent of training status and sports and of the extremities involved. Furthermore, we provided novel evidence that MoSlo can be modulated by means of anodal tDCS over M1 leg area in both trained athletes and NA. More precisely, we were able to induce a reduction in MoSlo specifically during FTT. These findings might be of interest for many sports where fatigability resistance is a limiting factor in maintaining repetitive movement patterns. Future studies should aim at transferring this knowledge into a context of sport-specific training and examine long-term tDCS-effects on sports performance.

## Data Availability

The datasets used and/or analysed during the current study are kept in the Institute for General Kinesiology and Exercise Science, University of Leipzig and are available from the corresponding author on reasonable request.

## Abbreviations

ANOVA: Analysis of variance
BF: Bayes factor
CNS: Central nervous system
CSF: Cerebrospinal fluid
EEG: Electroencephalography
EMG: Electromyography
FB: Football players
FL: Foot left
fMRI: Functional magnetic resonance imaging
FR: Foot right
FTT: Foot tapping task
GM: Gray matter
HB: Handball players
HL: Hand left
HR: Hand right
HTT: Hand tapping task
JASP: Jeffreys’s Amazing Statistics Program
LQ: Laterality quotient
M1: Primary motor cortex
MEP: Motor evoked potential
MoSlo: Motor slowing
MVC: Maximal voluntary contraction
MVR: Maximal voluntary rate
NA: Non-athletes
PMC: Premotor cortex
PMd: Dorsal premotor cortex
SD: Standard deviation
SE: Standard error
SM1: Primary sensorimotor cortex
SMA: Supplementary motor area
tDCS: Transcranial direct current stimulation
TMS: Transcranial magnetic stimulation
WM: White matter
$\eta_{p}^{2}$: Partial eta-squared
